# The role of barrier membranes for guided bone regeneration and restoration of large bone defects: current experimental and clinical evidence

**DOI:** 10.1186/1741-7015-10-81

**Published:** 2012-07-26

**Authors:** Rozalia Dimitriou, George I Mataliotakis, Giorgio Maria Calori, Peter V Giannoudis

**Affiliations:** 1 Department of Trauma and Orthopaedics, Academic Unit, Clarendon Wing, Leeds Teaching Hospitals NHS Trust, Great George Street, Leeds LS1 3EX, UK; 2 Department of Trauma and Orthopedics, University of Milan, Orthopedic Institute, G. Pini, University of Milan, Italy; 3 Leeds NIHR Biomedical Research Unit, Leeds Institute of Molecular Medicine, Beckett Street, Leeds, LS9 7TF UK

**Keywords:** bone regeneration, bone defect, barrier membranes, non-resorbable membranes, bioresorbable/absorbable membranes

## Abstract

Treatment of large bone defects represents a great challenge in orthopedic and craniomaxillofacial surgery. Although there are several methods for bone reconstruction, they all have specific indications and limitations. The concept of using barrier membranes for restoration of bone defects has been developed in an effort to simplify their treatment by offering a sinlge-staged procedure. Research on this field of bone regeneration is ongoing, with evidence being mainly attained from preclinical studies. The purpose of this review is to summarize the current experimental and clinical evidence on the use of barrier membranes for restoration of bone defects in maxillofacial and orthopedic surgery. Although there are a few promising preliminary human studies, before clinical applications can be recommended, future research should aim to establish the 'ideal' barrier membrane and delineate the need for additional bone grafting materials aiming to 'mimic' or even accelerate the normal process of bone formation. Reproducible results and long-term observations with barrier membranes in animal studies, and particularly in large animal models, are required as well as well-designed clinical studies to evaluate their safety, efficacy and cost-effectiveness.

## Introduction

Treatment of large bone defects represents a great challenge, as bone regeneration is required in large quantity and may be beyond the potential for self-healing. Large bone defects include segmental or large cortical defects created by trauma, infection, tumor resection, aseptic loosening around implants and skeletal abnormalities [1,2]. Critical size defect (CSD) is defined as the defect with the minimum length that cannot be spontaneously bridged leading to non-union [2,3]. Such defects are generally accepted to be ≥ 1.5 to 2 times the diameter of the long bone diaphysis, but they vary according to the host and the bone [2].

Although many methods for bone reconstruction exist, they all have specific indications and limitations. Established methods are distraction osteogenesis and bone transport, or bone grafting, including autologous bone grafts, bone marrow aspirate, allografts, bone substitutes or growth factors [4-8]. Furthermore, the concept of an induced-membrane represents another strategy for bone regeneration and particularly in cases of large bone defects secondary to trauma, infection or tumor excision. This method involves a two-stage procedure, where a 'biological' membrane is induced as a foreign body response after application of a cement spacer at the first stage, acting as a 'chamber' for the insertion of autologous bone-graft at the second stage [9-11]. It has been shown that this induced membrane possesses osteoinductive, osteogenic and angiogenic properties and several clinical studies have demonstrated satisfactory results [9,12]. Finally, the concept of Guided Bone Regeneration (GBR) using a bioabsorbable or non-resorbable membrane that acts as a barrier to prevent soft-tissue invasion into the defect and forms a 'chamber' to 'guide' the bone regeneration process [13-15] is also used for bone reconstruction.

Historically, the concept of GBR has been used in experimental reconstructive surgery since the mid-1950s, for spinal fusion [16] and maxillofacial reconstruction [17,18]. The initial hypothesis was that different cellular components in the tissue have varying rates of migration into a wound area during healing and that a mechanical hindrance would exclude the invasion of inhibiting substances, such as fibroblasts [19]. Preliminary studies showed that the use of a non-resorbable membrane as a mechanical barrier resulted in complete healing of the bone defect *in vivo *[20], and collagen membranes prevented the apical migration of epithelium and supported new connective tissue attachment and tissue regeneration [21]. The regeneration process occurring within the barrier membrane involves angiogenesis and migration of osteogenic cells from the periphery towards the center to create a well-vascularized granulation tissue. Initial organization of the blood clot is followed by vascular ingrowth and woven bone deposition, subsequent lamellar bone formation and finally remodeling, resembling bone growth [22,23]. When ingrowth of bone marrow into the bone defect was hindered or delayed, regeneration of mineralized bone was also delayed [24]. However, in large defects, bone formation occurs only to the marginal stable zone with a central zone of disorganized loose connective tissue, and, therefore, additional use of bone-graft materials is required in these cases, with the graft acting as a scaffold for osteoconduction and as a source of osteogenic and osteoinductive substances for lamellar bone formation [23].

### Types of barrier membranes, their basic characteristics and specific considerations

Although different non-resorbable and bioresorbable barrier membranes have been developed and their use has been extensively investigated, research is ongoing to develop the 'ideal' membrane for clinical applications. The basic characteristics of these membranes are biocompatibility, cell-occlusiveness, space-making, tissue integration, and clinical manageability [15,25].

Non-resorbable membranes and especially expanded-polytetrafuoroethylene (e-PTFE, Teflon) have been extensively studied [26]. They are biocompatible and maintain their structural integrity during implantation. They have superior space-maintaining properties and capacity for cell occlusion than degradable membranes, as the latter tend to collapse depending on the size of the defect [27]. Other non-resorbable membranes are titanium reinforced ePTFE, high-density-PTFE, or titanium mesh mainly used in oral and maxillofacial surgery [23] (Table 1). Semipermeable ePTFE is more effective than the high-density ePTFE with respect to bone regeneration [28]. For bone regeneration of large segmental bone defects, the cylindrical titanium mesh cage has been used as a scaffold with satisfactory preliminary results [29]. However, a second surgical procedure is required for removal, which represents a limitation and involves a potential risk to the newly regenerated tissues [30]. Finally, membrane exposure is frequent, increasing the risk of secondary infection [31,32].

**Table 1:** Summary of the different types of barrier-membranes used for reconstruction of bone defects

Types of membranes			
**Bioresorbable membranes**		**Advantages**	**Disadvantages**

Natural membranes	Collagen (different subtypes, predominantly type-I collagen, derived from different animals, (bovine or porcine) and from different sites (tendon or dermis) [40]	- highly biocompatible (no adverse effect to surrounding tissues during degradation) - it promotes wound healing [41] - it allows good integration with connective tissue (fibrous encapsulation with differentiation of a periosteum-like tissue upon the external bony surface) [42,43] - osteoblasts and fibroblasts can attach to collagen membranes irrespective of its origin [44] - differently cross-linked collagen membranes can promote cell attachment and proliferation [45]	- degradation *in vivo *is too rapid to maintain the structural integrity necessary for bone regeneration [44] - different cross-linking techniques used to prolong degradation time (it varies from four weeks up to six months) [40,41,46] - differently cross-linked collagen membranes can also inhibit cell attachment and proliferation [45] - chemicals used for cross-linking have cytotoxic effects on the surrounding tissues leading to gap formation between the membrane and the connective tissue and facilitate microbial accumulation [43] (to address this, a non-chemical cross-linking nanofibrous collagen membrane has been developed) [47] - variable mechanical properties among the different available membranes - risk of peri-operative rupture - moistening of the membrane (unavoidable *in vivo*) alters considerably the mechanical properties [48] - possible disease transmission from animals to humans [21,31]
	
	Chitosan or chitosan-collagen hybrid	- non-toxic natural polymer (polysaccharide) - it enhances wound healing and bone formation [49] - it has hemostatic properties [50] - excellent biocompatibility [51], osteogenic cells can proliferate and express osteogenic markers [51] - chitosan-hybrid membranes have superior mechanical properties [52,53]	- limited evidence from *in vivo *studies

Synthetic membranes	Aliphatic polyesters: PLLA, PLGA, polydioxanone and their co-polymers [52-54]	- the most commonly used and studied bioabsorbable polymer - commercially available and approved for clinical use - by changing the composition and the manufacturing procedure, resorption time, handling properties and mechanical durability can be adjusted to suit the clinical situation [54] - different chemical compositions did not affect on bone regeneration *in vivo *[55] - slow-degrading membranes induce greater amounts of neovascularization and a thinner fibrous capsule versus fast degrading membranes [56]	- they can induce host-tissue response and foreign body reactions during degradation (by non-enzymatic hydrolysis) [13,38,42,57-59] - the moderate cytotoxic reactions may reduce cellular adhesion [43]

**Non-resorbable membranes**			

Expanded polytetrafuoroethylene (e-PTFE) And others: titanium reinforced ePTFE, high-density-PTFE, or titanium mesh [23]		- extensively studied [26] - biocompatible - they maintain their structural integrity during implantation and have superior space-maintaining properties and capacity for cell occlusion than degradable membranes - semipermeable ePTFE is more effective than the high-density ePTFE [28] - for large segmental bone defects, cylindrical titanium mesh cage used as a scaffold [29]	- a second surgical procedure is required for removal (additional potential risk to the newly regenerated tissues [30]) - membrane exposure is frequent, increasing the risk of secondary infection [31,32] - e-PTFE can induce slight to moderate cytotoxic reactions and reduce cellular adhesion

**Novel membranes**			

Alginate membrane		- close assimilation to bone surface - no inflammatory response [60] - easy handling with an alginate base self-setting barrier membrane versus a ready-made membrane [61] -more efficacious versus collagen membranes for mandibular and tibial defects [62,63]	- limited evidence from *in vivo *studies
	
Others [64-68]: - degradable biopolymer poly (lactide-co-ε-caprolactone)(PLCL), - a nano-hydroxyapatite/polyamide(nHA/PA66) composite - an *in situ*-formed polyethylene-glycol-hydrogel membrane - amniotic membranes - a bacterially-derived polymer - a hybrid membrane consisting of layers of collagen containing hydroxyapatite (HA) and chitosan [69] - polyethersulfone (PES) electrospun nanofibrous membranes [70] - a biomimetic tubular calcium phosphate (CaP)-coated nanofiber mesh combined with platelet rich plasma-mediated delivery of BMP-7 [71] - Latex [72] - membranes with additional anti-bacterial properties or antimicrobial coating [73-75]		- optimized properties for GBR - improved three-dimensional structure and osteogenic bioactivity - they can be loaded with cells to mimic natural bone - no foreign body inflammatory reaction or rejection and satisfactory bone formation - membranes with additional anti-bacterial properties or antimicrobial coating may reduce membrane-associated infections	

BMP, bone morphogenetic protein;GBR, guided bone regeneration; PLGA, poly(L-lactide-co-glycolide); PLLA, poly(L-lactide).

Bioresorbable membranes have been developed to avoid the need for surgical removal. Such membranes have been extensively studied, mainly in animals but also in humans in maxillofacial, regenerative periodontal, and neuro-surgery [14,33-38]. Recently, commercially available bioresorbable membranes have also been used for reconstruction of long bone defects in the clinical setting. It has been shown that they enhance bone healing, especially in cases with bone defects > 4 to 5 cm or with significant associated soft-tissue loss, where autologous bone grafting alone is not recommended due to risk of resorption [39], and they also secure the grafting material [31]. There are two broad categories of bioresorbable membranes: the natural and the synthetic membranes. Natural membranes are made of collagen or chitosan, whereas synthetic products are made of aliphatic polyesters, primarily poly(L-lactide) (PLLA) and poly(L-lactide-co-glycolide) (PLGA) co-polymers [23]. Overall, their advantages are: 1) they allow for a single-step procedure, 2) the shape and volume of the regenerated bone can be predefined-prefabricated, 3) they are radiolucent allowing imaging, and 4) their bioresorption eliminates potential effects of stress shielding of the regenerated bone. Conversely, there is variability and lack of control over the rate of membrane resorption, which is influenced by factors such as the local pH and material composition. A summary of the main characteristics, advantages and disadvantages of the different bioresorbable membranes is presented in Table 1[13,21,31,38,40-59]. Currently, mainly PLLA membranes are available for clinical use in orthopedic surgery; whereas PLLA, collagen and ePTFE membranes are used for GBR in maxillofacial, dental and neuro- surgery.

Although a number of barrier membranes are already being used in clinical practice, novel membranes have been developed in an effort to overcome the limitations of the currently used membranes. Such novel membranes include alginate membranes, new degradable co-polymers, hybrid or nanofibrous membranes, as well as amniotic membranes. They are summarized in Table 1[60-75]. Ongoing research is evaluating these novel membranes, aiming to establish an 'ideal' membrane for bone regeneration with optimized characteristics in terms of biocompatibility, space-making, tissue integration and clinical manageability for maximum clinical efficacy and safety.

### The role of porosity and topography of the barrier membranes

The pore size of the barrier membrane is very important in order to prevent excessive penetration of fibrous tissue into the bone defect (soft tissue ingrowth) but to allow neovascularization and bone formation. Differences in the intensity of bone regeneration were observed depending on the pore size [76]. Pores in excess of 100 μm are required for the rapid penetration of highly vascular connective tissue, and small pores tend to become filled with more avascular tissue [77], as they are inadequate for penetration of capillaries [78]. A pore size of 50 to 100 μm allows bone ingrowth, but size greater than 150 μm is required for osteon formation [79,80]. A recent animal study showed that macroporous membranes facilitated greater bone regeneration compared to microporous membranes and prevented significant soft-tissue ingrowth [81]. Further research should be directed to identify the critical pore size, since an increase in pore size may result in decreased mechanical properties. A multilayer scaffold has been suggested to achieve suitable mechanical properties and porosity and mimic the structure of cancellous and cortical bone [82]. In addition to the porosity, the tri-dimensional topography of the membrane with interconnecting pores and channels is also important, as it can alter the cell occlusion properties and the biologic response of different cell types to the membrane [83].

### The role of soft tissue ingrowth

Although barrier membranes are used to prevent soft-tissue invasion, a thin layer of soft-tissue ingrowth (up to 1 mm thickness) can be formed under the membrane, overlying the regenerated bone [84-86]. This may be secondary to shrinkage of the initial blood clot under the membrane, entrapment of air or membrane micromovements. Currently, it is not known if this soft-tissue layer under the membrane undergoes mineralization if left for a long period. Some studies reported this tissue-layer was a periosteum-like tissue, and others reported it to be fibrous tissue [81] but its clinical implications are unknown.

### The role of mechanical stability

It is known that micromovements between bone and any implanted material prevent bone formation, resulting in the development of fibrous tissue [87,88]. Adequate stability and minimal stress are required to allow the early tissue that infiltrates through the pores to differentiate into bone by direct or appositional bone formation [81]. Bone formation can occur within porous materials even with limited initial movement provided the site is highly vascular and local inflammatory reaction is minimal [89]. New vascular network formation, which is a prerequisite for bone formation, is also highly sensitive to mechanical conditions with delayed mechanical loading significantly enhancing bone formation and stimulating vascular remodelling by increasing the number of large vessels and decreasing the number of small vessels [90]. Therefore, optimal stability should be provided in terms of the attachment of the membrane itself, since most bioresorbable membranes are flexible and they cannot be applied without additional fixation as well as the type of fixation of the bone defect [91]. To maximize stability of the membrane, the use of membrane-fixing pins has been suggested. It has been observed that bone formation is significantly enhanced when the resorbable membrane is tightly attached and immobilized to the bone surface [92]. Regarding the effect of the type of additional fixation to the process of bone formation, it is known that intermediate tissues, such as fibrous tissue, cartilage and woven bone, precede final bone formation, with the mechanical loading affecting the regeneration process and different stress distribution favoring or inhibiting differentiation of particular tissue phenotypes [93]. High shear strain and fluid flows stimulate fibrous tissue formation, whereas lower levels stimulate formation of cartilage, and even lower levels favor ossification. It has been demonstrated *in vivo *that there is more rapid and more organized new bone formation in rigidly fixed defects with plate osteosynthesis, covered with a resorbable collagen membrane, compared to non-rigidly fixed defects [94].

### Literature Review

As research on the field of bone regeneration is ongoing and the evidence is expanding, we aimed to summarize the current experimental and clinical research on the use of barrier membranes for restoration of bone defects and focus on maxillofacial and orthopedic applications. We searched the PubMed Medline and Ovid Medline databases, from 1991 to 2011, to retrieve all relevant articles reporting on the use of absorbable and/or non-absorbable membranes for bone regeneration in animal and clinical studies. Different combinations of searching terms were used including: membrane/bone regeneration/long bone/bone defect/segmental bone defect/segmental mandibular defect/mandibular defect. The search was restricted to studies published in English. We analyzed all preclinical studies using established animal models to evaluate barrier membranes for bone regeneration of segmental, large and critical-sized mandibular or long-bone defects, in which bone regeneration was documented and assessed using radiological or biomechanical and/or histological analysis. Regarding the clinical studies, all papers reporting on the clinical use of barrier membranes were analyzed.

The majority of studies were preclinical and the clinical studies were mainly retrospective case series. The summaries of the studies are shown in Tables 2 to 7.

**Table 2:** Summary of studies using membranes for segmental mandibular defects in small animal models

Author/Year [ref]	Animal model	Type of membrane	Study design	Assessment of bone regeneration	Outcome
Kazakos 2011 [95]	Rabbits mandible	platelet-rich plasma (PRP) gel alone or human fascia lata membrane (HFL)	Group I: HFL Group II: PRP gel Group III: HFL+PRP	Histological at 12 weeks	None of the control sides and the PRP treated sides had full development of bone or filling of the defect through bone bridging. The application of PRP gel alone or in combination with HFL does not seem to enhance bone regeneration.

Kim 2011 [64]	Rats mandible	a novel nanofibrous membrane of a degradable biopolymer poly (lactide-co-ε-caprolactone) (PLCL)	A 5 mm critical-sized defect	Histological at four weeks	The assessment of cell compatibility showed favorable cell adhesion and growth on the nanofiber PLCL membrane. At four weeks, the PLCL nanofibrous membrane induced better guided new bone formation than the defect control group while protecting the bone defect against the ingrowth of fibrous tissues.

Hoogeveen 2009 [96]	Rats mandible	a degradable membrane of poly(DL-lactide-epsilon-caprolactone) (PDLLCL) versus collagen versus polytetrafluoroethylene (ePTFE)	Defects covered with a membrane (PDLLCL, collagen, or expanded ePTFE) or left uncovered (control).	At 2, 4 and 12 weeks using transversal microradiography	For defect closure and bone thickness all membrane-treated groups showed effect modification between time and membrane; these effects were more significant and larger in the collagen and ePTFE groups. In the non-treated controls no effect modification was observed. The membrane groups showed significantly better results than the control groups. The ePTFE and collagen membranes performed equally well and better than the PDLLCL membrane during this experiment. PDLLCL membrane not suitable for clinical application in its current form.

Gielkens 2008 [31]	Rats Mandible	a novel degradable synthetic membrane (Vivosorb) of poly(dl-lactide-epsilon-caprolactone) (PDLLCL) versus collagen and ePTFE membranes	A standardized 5 mm circular mandibular defect Four groups (control/uncovered, PDLLCL, collagen, ePTFE).	At 2, 4, and 12 weeks Microradiography and muCT	Bone formation was progressive when the defect was covered with a membrane. More bone formation was observed underneath the collagen and ePTFE membranes than the PDLLCL membranes. Bone formation in PDLLCL-covered defects was less and the high variation in the PDLLCL samples at 12 weeks may be caused by the moderate adherence of this membrane to bone compared with collagen.

He 2008 [97]	Rabbits mandible	a novel calcium alginate film (CAF) versus conventional collagen membrane (CM)	Bilateral critical size 5 mm mandibular defects covered with CAF (experimental group) or conventional collagen membrane (CM) or left empty (control group)	At one, two, four, six and eight weeks. Morphological and histomorphometric evaluation	The CAF guided early bone growth and appeared more effective as a bioabsorbable GTR membrane than CM. A significantly greater percentage of newly generated bone in CAF defects than that in CM defects and empty defects from two to six weeks post-operation. At six and eight weeks, significantly more mature lamella bone had formed with CAF than with CM.

Thomaidis 2008 [34]	Rabbit mandible	five different membranes: - HFL (human fascia lata) - HP (human pericardium) - HFT (human fascia temporalis) - BP (bovine pericardium), - e- PTFE	9-mm circular mandibular defects were created bilaterally. Five groups for each membrane and the defect on the other side served as a control.	Histological at ten weeks	Membranes were significantly superior to the controls. HFL, HP, BP, and PTFE were significantly superior to HFT HFT is not recommended for GBR techniques for osseous defects beyond the critical size.

Asikainen 2006 [54]	Rabbit mandible	poly(desaminotyrosyl-tyrosine-ethyl ester carbonate) (PDTE carbonate) membrane (thickness 0.2-0.3 mm)	A through-and-through defect (12 × 6 mm). Group 1: defects left unfilled but covered with membrane Group 2: defects filled with bioactive glass mesh and covered with membrane Controls were left uncovered and unfilled.	Histological at 6, 12, 24 and 52 weeks	PDTE carbonate elicited a modest foreign body reaction in the tissues, which was uniform throughout the study. New bone formation was seen in all samples after six weeks. Group 1 had more new bone formation until 24 weeks and after this the difference settled. PDTE carbonate membranes have good biocompatibility and are sufficient to enhance bone growth without additional supportive matrix.

Jianqi 2002 [63]	Rabbit mandible	calcium alginate film (CAF) with CM	Circular bone defects with 5-mm diameter one side were covered with a CAF, and the contralateral side with CM.	gross, radiographic, electromicroscopic, histologic, and immunohistochemical analyses and image pattern analysis system at one, two, four, six, and eight weeks	CM absorbed more slowly but collected fewer osteoinductive factors (P < .05) in the early period. CAF induced dense bone formation, whereas CM produced less newly formed bone. CAF is more efficacious than CM in guided bone regeneration in this animal model.

Stetzer 2002 [94]	Rabbit mandible	collagen membrane	Bilateral critical size (4 mm) defects maxillary segments were rigidly or not rigidly fixed using bone microplates and screws or osteosynthetic wires. The defects were covered with a resorbable collagen membrane or left uncovered.	At four weeks serial radiographs and histologic/histomorphometric analyses	The rigidly fixed defects, covered with membrane, showed the most rapid and organized new bone formation. They averaged approximately 40% more new bone in the osteotomy site compared with the rigidly fixed defects with no membrane. No rigidly fixed defects with no membrane showed an ingrowth of fibroblasts and fibrous non-unions.

Zahedi 1998 [98]	Rats mandible	diphenylphosphorylazide-crosslinked type I bovine collagen membrane (DPPA)	5 mm diameter full-thickness circular bone defects one side covered by the membrane the other side uncovered (control)	Histological at 7, 15, 30, 90, and 180 days	Although at early stages of healing similar amounts of bone formation were observed in the both groups, after one month of healing, most of the experimental defects were completely closed with new bone, while in the control defects, only limited amounts of new bone were observed at the rims and in the lingual aspect of the lesions. In the 90- and 180-day animals, all experimental defects were completely closed, while in the control defects, no statistically significant increase in bone regeneration was observed.

Linde 1995 [99]	Rats mandible	e-PTFE membrane (GORE-TEX)	Circular transosseous 'critical size' defects in mandibles of rats were either implanted with recombinant human bone morphogenetic protein type 2 (rhBMP-2) or were left empty; half the number of implanted and half the number of empty defects were covered with the e-PTFE membrane	At 12 and 24 days of healing by a histomorphological scoring system	Implantation of rhBMP-2 alone resulted in bony bridging of the defect after only 12 days, but also in voluminous amounts of new bone outside the original defect area. When rhBMP-2 was combined with membrane, newly formed woven bone bridged the defect and the bone contour was maintained by the membrane. The combined treatment with membrane and rhBMP-2 demonstrated a significantly better bone healing than with e-PTFE membrane alone at both 12 days and 24 days of healing. RhBMP-2 had a strong osteoinductive potential and this potential was retained when combining the rhBMP-2 with the osteopromotive membrane technique, yielding better bone healing than with the membrane alone, and at the same time maintaining the bone contour.

Zellin 1995 [100]	Rats mandible	ten different biodegradable and non-biodegradable membrane materials	Standardized bilateral critical size mandibular defects and randomly covered with the different types of membrane	Scanning electron microscopy and histological analysis at six weeks	At six weeks, varying degrees of bone healing seen beneath the different membranes. Some of the membranes revealed a good osteopromotive effect, whereas others had little or no beneficial effects on bone healing, even if seemingly chemically closely related. Certain membrane materials caused a pronounced inflammatory response in the surrounding soft tissue, while others displayed a low inflammatory reaction.

Dahlin 1994 [101]	Rats mandible	e-PTFE membrane	Standardized through-and-through critical size defects (non-union) On one side of the jaw, the defect was covered both buccally and lingually with an expanded polytetrafluoroethylene (e-PTFE) membrane. On the other side no membrane was used.	Histological at six weeks	Complete healing with bone of the membrane-covered defects at six weeks. No cartilage was present in any of the specimens. At the control sites (no membrane), the amount of newly produced bone showed variations, most through defects revealing the presence of a remaining central portion of connective tissue.

Kostopoulos 1994 [102]	Rats mandible	a polyhydroxybutyrate resorbable membrane	A 2 × 3 mm defect the contralateral side: no membrane	Histological analysis from 15 days to 6 months	The histological analysis demonstrated increasing bone fill in the test specimens from 15 to 180 days, whereas only 35% to 40% of the defect area in the control sides was filled with bone after 3 to 6 months. Ingrowth of muscular, glandular and connective tissue was consistently occurring in the control defects during healing.

Sandberg 1993 [103]	Rats mandible	three types of bioabsorbable membranes (BAMs) of polylactic/polyglycolic acid copolymers with different absorption times and comparisons with e-PTFE membrane.	Standardized 5 mm critical size defects	Histological at 1 to 12 weeks	BAMs were well tolerated by the tissue, causing just a mild inflammatory reaction along the membrane surfaces as long as the material remained in the tissue. The BAMs were as efficient as e-PTFE membranes. Healing in conjunction with one type of BAM seemed to occur somewhat more rapidly. BAMs represent a valid alternative to e-PTFE membranes to improve bone regeneration.

GTR, guided tissue regeneration; muCT, micro-computer tomography.

### Animal studies

Tables 2 to 5 summarize the preclinical studies with non-absorbable or bioabsorbable membranes. There were 23 animal studies reporting on the use of membranes in maxillo-facial surgery for reconstruction of segmental or critical mandibular defects using small or large animal models (Table 2, 15 studies [31,34,54,63,64,94-103] and Table 3, 8 studies [22,104-110], respectively). Overall, the membrane-treated groups showed improved bone formation within the mandibular defects compared to the non-treated animals [22,96,98]. Differences in the rate of bone regeneration and the inflammatory response in the surrounding soft tissues were observed with different types of membranes [31,97,100].

**Table 3:** Summary of studies using membranes for segmental mandibular defects in large animal models

Author/Year [ref]	Animal model	Type of membrane	Study design	Assessment of bone regeneration	Outcome
Jégoux 2010 [104]	Dogs Mandible	collagen	Segmental defects after mandibulectomy using calcium phosphate ceramics and collagen membrane with a delayed bone marrow grafting (after two months, bone marrow injection)	At 16-weeks Histological and scanning electronic microscopic analysis and X-ray microtomographic analysis	Successful osseous colonization bridged the entire length of the defects. The good new bone formation at the center and the periosteum-like formation at the periphery suggest the osteoinductive role of the bone marrow graft and the healing scaffold role of the membrane.

Borges 2009 [105]	Dogs mandible	acellular dermal matrix (ADM) in comparison with a bioabsorbable synthetic membrane	Control group (bioabsorbable membrane made of glycolide and lactide copolymer) Test group (ADM as a membrane).	At 8 and 16 weeks, radiological evaluation At 16 weeks: Clinical measurements of the width and thickness of the keratinized tissue and histomorphometric analysis	ADM acted as a barrier in GBR, with clinical, radiographic and histomorphometric results similar to those obtained with the bioabsorbable membrane

Sverzut 2008 [106]	Dogs mandible	poly L/DL-lactide 80/20% membrane with different permeability patterns	10 mm segmental defects Mechanical stabilization and 6 treatment groups: control, BG alone (bone graft), microporous membrane (poly L/DL-lactide 80/20%) (Mi); Mi plus BG; microporous laser-perforated (15 cm2 ratio) membrane (Mip), and Mip plus BG.	Histological, histomorphometry and fluorescence microscopy at six months	BG protected by Mip was consistently related to larger amounts of bone versus other groups. No difference between defects treated with Mip alone and BG alone. Mi alone rendered the least bone area and reduced the amount of grafted bone to control levels. Bone formation was incipient in the BG group at three months regardless of whether or not it was covered by membrane. In contrast, GBR with Mip tended to enhance bone formation activity at three months. The use of Mip alone could be a useful alternative to BG. The combination of Mip membrane and BG efficiently delivered increased bone amounts in segmental defects compared with other treatment modalities.

Bornstein 2007 [107]	Dogs mandibles	two bioabsorbable collagen membranes: collagen membrane versus cross-linked collagen membrane (CCM).	three standardized defects filled with bone chips and deproteinized bovine bone mineral (DBBM), and covered by three different methods: control = no membrane; test 1 = collagen membrane; and test 2 = cross-linked collagen membrane (CCM). Each side of the mandible was allocated to one of two healing periods (8 or 16 weeks).	At 8 and 16 weeks Histomorphometric analysis	For all groups, the defect fill height increased between weeks 8 and 16. The CCM group showed a statistically significant increase over time and the highest value of all treatment modalities after 16 weeks of healing. The CCM showed a limited beneficial effect on bone regeneration in membrane-protected defects in dog mandibles when healing was uneventful. However, the increased complication rate with CCM requires a more detailed preclinical and clinical examination.

Zubery 2007 [108]	Dogs mandibles	type I collagen membrane (GLYM) using a novel cross-linking technology versus a non-cross-linked bilayer type I and III collagen membrane (BCM)	Mandibular bilateral critical size defects five groups: GLYM + bovine bone mineral (BBM), BCM + BBM, BBM alone, sham-operated, or GLYM alone.	At 8, 16, and 24 weeks, Qualitative, semiquantitative, and quantitative light microscopy analyses	Membrane-protected sites displayed bone filling between the BBM particles with almost complete restoration of the original ridge morphology that increased with time up to 16 weeks and remained unchanged at 24 weeks. Both membranes showed marked degradation within 16 to 24 weeks, with BCM inconsistency that was undetectable in one of four sites at 8, 16, and 24 weeks. Membrane ossification was observed in all GLYM sites and in only one BCM site, which progressed with time to 24 weeks. Bone increased by approximately 1 mm on the lingual side, where the GLYM membrane was in direct contact with bone.

Peled 2002 [109]	Dogs mandibles	titanium-reinforced expanded ePTFE membrane (ePTFE-TR)	Mandibulectomy defects (25 mm × 15 mm) ePTFE-TR or control (repositioning flaps)	At four to six months Macroscopic and histological/histomorphometric evaluation	The size of the residual defect in the experimental sites was much smaller compared to the controls, which was statistically significant. Histomorphometric measurements of new bone formation revealed a similar pattern. These differences were also statistically significant.

Fritz 2000 [110]	Macaca mulatta monkeys Mandibles	reinforced ePTFE membranes	Standardized 8 × 19 mm mandibular defects Reinforced ePTFE membranes held in place with mini screws and sutures for anywhere from 1 to 12 months. No material added to the defect.	Digital subtraction radiology and fluorescent labelling with tetracycline and histomorphometry	Data suggest that membranes left in situ for 1 month or less result in minimal bone gain compared with membranes left in place from 2 to 12 months. In addition, labelling and stained sections clearly showed that the bone produced after 2 months of membrane placement is mature.

Schenk 1994 [22]	Dogs mandibles	standard and prototype reinforced e-PTFE membranes	Standard and prototype reinforced e-PTFE membranes and control (no membranes)	At two and four months Histologic evaluation	Control sites without membranes exhibited incomplete osseous healing with a persisting defect. Test sites with membranes demonstrated significantly better bone healing, although bone regeneration was not yet completed at 4 months. Histologic evaluation showed that bone regeneration, once activated, progresses in a programmed sequence which closely resembles the pattern of bone development and growth.

A total of 27 animal studies reported on the use of membranes for reconstruction of long bone defects. There were 21 studies using a small animal model (Table 4) [55,60,62,76,82,111-126], and only six studies using a large animal model (Table 5) [127-132]. As in maxillofacial animal studies, superior bone healing has also been observed in long bones treated with a barrier-membrane compared to the non-treated defects using bioabsorbable as well as non-resorbable membranes [111,117,118,121]. Bone defects treated with improved bilayer membranes displayed better regeneration of cortical bone tissue [112], whereas novel composite membranes displayed affluent neovascularization and bone formation with little fibrous tissue formation [82]. The differences in chemical composition of the polylactide membranes did not seem to have an evident effect on bone healing in a small animal model [55], but different pore sizes resulted in differences in the intensity of the bone regeneration process [76]. Large animal studies also showed promising results for restoration of long bone defects but only when combined with additional bone grafting material [131,132]. When two concentric perforated membranes (the tube-in-tube implant) were used in combination with cancellous bone graft in segmental diaphyseal defects, a 'neocortex' was reconstituted with well-defined thickness [132].

**Table 4:** Summary of studies using resorbable membranes for long bone defects in small animal models

Author/Year [ref]	Animal model	Type of membrane	Study design	Assessment of bone regeneration	Outcome
Bernabé 2012 [111]	Rats tibia	decalcified cortical osseous membrane [GenDerm(^®^)]	To study the effect of using lyophilized bovine bone (GenOx(^®^) organic matrix) with (or without) GBR (using a decalcified cortical osseous membrane [GenDerm(^®^)]) Surgically created critical-size defects group I (control) group II (defect filled with GenOx(^®^) group III (defect covered by GenDerm(^®^) group IV (defect filled with GenOx(^®^) and covered by GenDerm(^®^)	At 30 or 90 days Histological and histomorphometrical	Superior bone healing in all groups compared to control group. Group IV showed evidence of more advanced healing at 30 and 90 days compared with the other groups.

Cai 2010 [112]	Rabbits tibia	electrospun PLLA nanofibrous membrane +/- collagen	Large bony defects Three groups: a nanofiber-reinforced bilayer membrane, a nanofibrous membrane, or a collagenous membrane alone	At three and six weeks Radiological and histological	Bilayer membrane group had more bony tissue formation at thre weeks. At six weeks, only the bilayer membrane-treated bone defects displayed better regeneration of cortical bone tissue. Other groups: defects filled with spongy bone-like tissue.

Lysiak-Drwal 2008 [113]	Rabbits femur	collagen	A 5 mm in diameter defect created transcutaneously Group I: control, left to heal spontaneously Group II (BOC+BG): filled with Bio-Oss Collagen and Bio Gide Perio membrane Group III: BOC and platelet-rich plasma	At one and three months Histological	Greater number of bone trabeculas after implantation in groups II and III compared to control.

He 2007 [62]	Rabbits tibia	calcium alginate film (CAF) versus collagen or no membrane	Circular bone 5 mm diameter defects CAF versus collagen versus no membranes	At one, two, four, six, and eight weeks Gross evaluation, radiological, histological, immuno-histochemical, and an image pattern analysis system	CAF induced dense bone formation, whereas CM induced less new bone, and the blank control sites even less.

Kong 2007 [82]	Rabbit fibula	chitosan membrane	5 mm defect filled with a porous nano-hydroxyapatite-chitosan composite multilayer scaffold	At 12 weeks Radiological and histological	Composite membranes are implanted into a fibular defect to evaluate the osteoconductivity and the efficacy as a barrier to fibrous tissue ingrowth: affluent blood vessels and bone formation found in the center of the scaffold and little fibrous tissue noted within the defect.

Gerbi 2005 [114]	Rats Femur	decalcified cortical osseous membrane [GenDerms]) +/- Laser irradiation	Surgical bone defects, five groups: Group I (control); Group II (Gen-ox: lyophilized bovine bone organic matrix) Group III (Gen-ox + Laser); Group IV (Gen-ox + Gen-derm); Group V (Gen-ox + Gen-derm + Laser)	At 15, 21, and 30 days. Histological assessment.	Improved amount of collagen fibers at early stages of the bone healing (15 days) and increased amount of well organized bone trabeculae at 30 days on irradiated animals compared to non irradiated ones.

Nasser 2005 [115]	Rabbits radius	two types: - non-resorbable ethyl cellulose membrane (EC, N-type, Hercules Inc., Delaware) - resorbable chitosan membrane (CH, poly(D-glucosamine), Aldrich)	1 cm segmental radial defect (2.5 times the radial bone diameter)	Radiological Every two weeks for an eight-week period. Bone density in the different osteotomy quadrants Histological evaluation (scoring system) Four rabbits at two week intervals	EC group: an increase in the new bone density was apparent in all quadrants during the first four weeks, followed by a sharp decline in bone density. CH group: different biological behavior, lesser increment in bone density in the first four weeks but continued throughout the eight weeks. Possible cause: degradation of membrane products and foreign body reaction. Based on histological findings: EC membranes are better osteoinducers. Radiological findings: CH membranes are better osteoconductors.

Moore 2004 [116]	Rats femur	fresh, morselized porcine small intestine submucosa (SIS) used as preformed tubular SIS grafts	Critical length segmental defects four groups: unfilled or filled with morselized cancellous bone, or spanned with intramedullary tubes or periosteal sleeves fabricated from SIS	Radiological (biweekly) At 12 weeks histological, and mechanical testing	New bone formation in all defects treated with cancellous bone. Fibrous tissue and no bone formation in defects left unfilled or treated with SIS SIS persisted at twelve weeks. Cellular response to SIS: mild mononuclear infiltrate in the loose or delaminated superficial layers of the tubes and sleeves, with few cells in the deeper layers. The ability of SIS to support or stimulate growth of bone across a critical length segmental bone defect is doubted.

Ip 2002 [117]	Rabbit radii	poly(L/DL-lactide) membrane or sponge (bioabsorbable)	Segmental defect, four groups: I: untreated + plaster, II: plate fixation III: membrane + plate fixation IV: sponge + plate fixation	Radiological At eight weeks histological	Group I + II: no healing Group III: healing Group IV: more abundant healing than III

Ueyama 2002 [60]	Rat tibia	alginate membrane (bioabsorbable)	Calcium chloride aqueous solution dropped into the bone defect, which is filled with sodium alginate aqueous solution.	n/a	Evaluation of short-term biocompatibility of alginate membrane. The healing process in bone defects covered with an alginate membrane was delayed in comparison with that of controls; however, the defect was restored to nearly original condition. In contrast, in the controls, bone defect repairs exhibited partitioning as a result of connective tissue involvement. A relation between the sodium alginate concentration and the rate of absorption of the sodium alginate membrane was noted. No inflammatory response around the alginate membrane.

Matsuzaka 2001 [118]	Rats tibia	ePTFE (non-resorbable)	e-PTFE groups control groups (without membrane)	Histological At six, eight, or ten days using immunohistochemistry and confocal laser scanning microscopy to investigate new bone mineralization	The bone occupation ratio increased day by day, but the experimental groups had significantly higher ratios than control groups (without membrane) at each of the time periods. More rapid mineralization in the experimental groups vrsus controls. GBR accelerates the migration of osteogenic cells, the formation of new bone, and mineralization in the defect created by the e-PTFE membrane.

Nyman 2001 [119]	Rabbits radius	ePTFE (non-resorbable)	10 mm diaphyseal defects (both sides: test and control) Test side: bone marrow ingrowth into the defects was hindered or delayed (plugging the opening of the cut bone ends with gutta-percha points; plugging with Gelfoam; or by removing the bone marrow by flushing with saline), in all defects: an ePTFE membrane, shaped as a tube	Regular radiological At four to five months, histological	Any attempts to delay or prevent bone marrow ingrowth into the defects retard regeneration of segmental long-bone defects.

Caiazza 2000 [120]	Rabbits femur	+/- resorbable collagen membrane (bovine Achilles tendon collagen Type I)	Ten hydroxyapatite-coated titanium fixtures inserted within a created cortical defect, covered with a resorbable membrane Control: no membrane	At 60 days Tensile shear-stress at break testing and histological	Lower performance without membrane Neoformed cortical bone present cervically around implant was much thicker when a collagen membrane was used.

Gogolewski 2000 [55]	Rabbits radius	poly(L/D-lactide) and poly(L/DL-lactide) membranes (bioabsorbable)	10 mm diaphyseal segmental defects - membranes from poly(L/D-lactide) - poly(L/DL-lactide) to determine whether chemical composition of the membrane affected the bone healing in the defect. Control: previous study with same animal model and similar defects not covered with membranes or covered with poly(L-lactide) membranes	Radiological at two, four, six, and eight weeks Histological at 3, 6 and 12 months	At one year: complete bone regeneration in the defects covered with the poly(L/D-lactide) membrane, only one animal with no regeneration and one animal with pseudarthrosis. Complete bone regeneration in all animals for the poly(L/DL-lactide) membrane (one animal died during surgery). The quality of the interface between the new bone and the membrane seemed to be affected by the chemical structure of the polylactides used for membranes preparation. The differences in chemical composition of the polylactide membranes did not have an evident effect on the bone regeneration process in segmental defects of the rabbit radii.

Ishikawa 1999 [121]	Rats tibia	alginate membrane	3 mm × 10 mm bicortical bone defect filled with 0.5, 1.0, or 1.5% Na-Alg aqueous solution, then 3% calcium chloride aqueous solution was dropped on the Na-Alg solution to form an alginate membrane. four groups: (a) control group (no solutions) (b) alginate membrane with 0.5% Na-Alg solution (c) alginate membrane with 1.0% Na-Alg solution (d) alginate membrane with 1.5% Na-Alg solution	Histological At four weeks	Control group: bone defect filled with connective tissue. 0.5% Na-Alg solution: part of the alginate membrane had disappeared and connective tissue had begun to grow in the bone defect. 1.0 or 1.5% Na-Alg solution: the alginate membrane prevented any ingress of connective tissue to the bone defect, and the bone defect was reconstructed with new bone. At this stage, the alginate membrane still was observed, and the amount of unabsorbed alginate was larger for higher concentrations of Na-Alg aqueous solution. No inflammatory response was observed around the alginate membrane.

Suckow 1999 [122]	Rats radius	small intestinal submucosa (SIS)	The defect was either left unfilled or implanted with SIS, demineralized cortical bone (DMCB), or ovalbumin.	Radiographically and histologically after 3, 6, 12, and 24 weeks.	Tissue remodelling within the defect was evident by week three in SIS- and DMCB-treated rats. Filling was characterized initially by infiltration of mononuclear cells and extracellular material in SIS-implanted rats and multifocal remodelling bone particles and cartilage formation in DMCB-implanted rats. Cartilage was observed as early as three weeks and bone as early as six weeks in SIS-implanted rats. Filling of the defect arose from multiple foci in DMCB-implanted rats, but was contiguous with and parallel to the ulnar shaft in SIS-implanted rats, suggesting that defect repair by SIS may be conductive rather than inductive. Rats in which the defect was left unfilled demonstrated slow but progressive filling of the defect, characterized by mononuclear cell infiltrates and fibrous extracellular material. SIS facilitated rapid filling of a long-bone defect.

Meinig 1997 [123]	Minipigs radius	polymer membranes: poly(L/DL-lactide) and poly(D-lactide) (bioabsorbable)	2.5 to 3 cm mid-diaphyseal defect five groups: (a) poly(L/DL-lactide), (b) poly(L/DL-lactide)-CaCO3, (c) poly(D-lactide), (d) poly(D-lactide)-CaCO3, (e) untreated defect Ulna left intact and no adjunctive internal or external fixation	Radiological (biweekly) At 12 weeks, histologic and microradiographic evaluation	The bone defects covered with membranes were completely reconstituted by six to eight weeks. Untreated defects healed with less bone formation and in a more disorganized pattern. Histologic evaluation of the implants demonstrated that the entire lumen of the implant was filled with bone, with some periosteal bone formation occurring on the outer surface of the membrane. Direct apposition of bone onto the membrane surface or minimal fibrous tissue interposition between membrane and new bone. No foreign body or adverse reaction to the membrane.

Lu 1996 [124]	Rabbits radius	silicone membrane	10-mm defect on radius silicone membrane sutured as a tube 10-mm defects were also produced on the control sides.	At 12 weeks radiological, three-point bending test, and histological	By the 12th week, seven of ten experimental sides were healed, two were healed with a connective cartilage zone, and one was not healed. None of the control was healed but the defect was occupied by soft tissue.

Pineda 1996 [76]	Rabbits radius	poly(L-lactide) membranes of various pore sizes (microporous, medium pore size (10 to 20 microns), and large pore size (20 to 200 microns) (bioabsorbable)	10 mm diaphyseal defect No internal fixation (assumption that the intact ulna splints the radius adequately)	Radiological at two and four weeks and six months	Bone regeneration in the majority of cases, regardless of pore size. Some differences in the intensity of the bone regeneration process. At two weeks, bone formation seen in all animals, but at six months five rabbits of five, four rabbits of five, and three rabbits of five implanted respectively with microporous, medium pore-size and large pore-size membrane showed complete regeneration of the defect.

Nyman 1995 [125]	Rabbits radius	ePTFE membrane (non-resorbable)	7 to 10 mm segmental diaphyseal defects Study group: membrane formed as a tube Control: contralateral side with no membrane	Radiological (obtained repeatedly) and histological at 13 or 27 weeks	Control group: some early subperiosteal callus formation and non-union of the defects at six weeks. Study group: subperiosteal bone formation at the bone ends first observed at two weeks. At nine weeks, a thin cortical bone bridged the defect along the inner surface of the membrane. Histology: an interrupted line of thin, cortical bone was observed along the inner surface of the barrier membrane. Fatty bone marrow occupied the central and largest volume of the defect.

Farso Nielsen 1992 [126]	Rabbits radius	polyurethane membrane (bioabsorbable)	1 cm segmental, osteoperiosteal defects Study group: membrane formed as a tube versus untreated control	At five weeks Radiological and histological	Controls: 90% non-union Membrane-treated defects: all healed by forming callus external to the membrane fusing the bone fragments. Loose connective tissue was predominant in the bone gap underneath the membrane.

**Table 5:** Summary of studies using resorbable membranes for long bone defect reconstruction in large animal models

Author/Year [ref]	Animal model	Type of membrane	Study design	Assessment of bone regeneration	Outcome
Rhodes 2010 [127]	Dogs humerus	Hyaluronan (Hyalonect)	Periosteal reconstruction of bone defects filled with a variety of conventional bone filling compounds.	Histological at six weeks	Hyalonect was shown to allow the regeneration of bone within the humeral defects while preventing fibrotic tissue in-growth, and allowing regeneration of tissue which, by six weeks, had begun to resemble natural periosteal tissue.

Oh 2006 [128]	Dogs humerus	betatricalcium phosphate and poly L-lactide-co-glycolide-coepsilon- caprolactone (TCP/PLGC)	Partial bone defects (length: a quarter of the full length of humerus, width: a quarter of middle diameter of the lateral aspect of humerus) Control group: the contralateral humerus	Computed tomography (CT) at four and eight weeks and histological	The result suggested that TCP/PLGC membrane is a good guided bone regeneration material to restore the original morphology of humerus in partial defect.

Beniker 2003 [129]	Pig femur	acellular dermal matrix (GraftJacket Acellular Periosteum Replacement Scaffold)	Segmental bone defect	Histological at six weeks	The scaffold protects the bone defect site as revealed by new bone formation within the margins of the defect and adjacent to the scaffold has been shown. Minimal to no soft tissue invasion into the defect site. Dermal membrane material may be used as a scaffold for periosteum regeneration by allowing for cellular repopulation, revascularization, and bone defect restoration.

Gerber 2002 [130]	Sheep Tibia	bioabsorbable polylactide membranes (L/DL-lactide) (80%-20%)	7-cm diaphyseal defect Resorbable polylactide membranes +ABG or a vascularized periosteal flap. four groups (fixation with a nail)	Clinical + post-mortem observation, radiological post-op and then weekly until week 16.	Polymeric membranes of adequate composition and pore size combined with ABG or vascularized periosteum allow for rapid and stable defect regeneration.

Gugala 2002 [131]	Sheep tibia	bioabsorbable polylactide membranes, with or without perforations, Single or double-tube designs	six groups: Polylactide membranes Single or double-tube designs +/_ cancellous bone grafting	Radiological (X-rays and CT) and histological at 16 weeks.	In groups without bone grafting non-union developed and persisted until 16 weeks. Defect healing was only observed when ABG was used along with the single or double microporous-perforated membranes. (new bone formation by 'creeping substitution' of the graft)

Gugala 1999 [132]	Sheep tibia	poly(LDL-lactide)	4 cm diaphyseal segmental defects 1) a single microporous membrane 2) a microporous internal membrane, and a membrane on the outer surface of the cortex (external membrane) 3) an external membrane laser-perforated (800 to 900 micrometers openings) 4) ABG and a single perforated membrane 5) one perforated internal membrane into the medullary cavity and another membrane on the outer surface of the cortex 6) as Group 5 + ABG between the two membranes	Radiological and histological	No bone healing in Groups 1, 2, 3, and 5. Only in Groups 4 and 6 the defects healed. In Group 4, new bone was dispersed across the 'medullary canal' formed by the membrane. In Group 6, the new bone had grown into the space between the outer and inner membranes, forming the 'neocortex'. The resorbable polymeric implant consisting of two concentric perforated membranes (the tube-in-tube implant) used in combination with cancellous bone graft to treat segmental diaphyseal defects allows for the reconstitution of the 'neocortex' with well-defined thickness.

### Clinical studies

Tables 6 and 7 summarize the clinical studies, in which absorbable membranes were used for bone regeneration of the mandible and the long bones, respectively. The absorbable membranes used were either experimental materials [57,133], similar to the ones used in the animal studies, or commercially available material manufactured for other purposes [13,134].

**Table 6:** Summary of clinical studies with bioresorbable membranes for reconstruction of segmental mandibular defects

Author/Year [ref]	Study design	No. of pts	Mandibular reconstruction	Defect size	Etiology	Type of membrane	Graft	Type of fixation	Outcome
Kinoshita 2004 [33]	case series (1995 to 2001)	62	Mandibulectomy (segmental defect and hemimandibulectomy)	_	malignant (22) and benign (30) tumors, cysts (5), osteomyelitis (2), alveolar atrophy (1) trauma (2)	Absorbable PLLA mesh	Autologous cancellous bone graft + bone marrow	+/- stainless steel wires	At six months post-operation: Excellent (markedly effective) 56.5% Good (effective) 27.4% Poor (not effective) 16.1% X-ray of the regenerated bone: 0 to 10% bone resorption in 31 cases 10% to 20% in six cases 20% to 30% in one case
			
Kinoshita 2000 [133]	case series (1995 to 1998)	41	Segmental defect or large partial defects mandibulectomy	_	malignant (19) and benign (22) tumors			-	Excellent: 19/41 (46.3%) Good 13/41 (31.7%) Poor 9/41 (22.2%) (local infection) 86.5% Success rate Five years: no problems from PLLA and good osseointegration
			
Kinoshita 1996 [57]	case series	2	Segmental defect or large partial defect	right to left molar areas	tumor			stainless steel wires	At three months: full bone regeneration

**Table 7:** Summary of clinical studies with bioabsorbable membranes for reconstruction of long bone defects

Author/Year [ref]	Study design	No of patients	Site	Size of defect	Etiology	Type of membrane	Graft	Type of fixation	Evaluation	Outcome
Meining 2010 [13]	case series	Six	Tibia (five) Femur (one)	≥ CSD	Trauma	poly(L/DL-lactide) 80/20% membranes with 50 to 70 μm pore size	RIA, ICBG	IMN, plating	X-rays	Healing one case: re-grafting after 15 months
		
Ip 2004 [134]	case series	Ten	Not reported	≤ 6 cm	Benign tumor, Osteomyelitis and trauma		polymeric scaffolds (sponges, 450 to 700 μm pore size) impregnated with bone marrow	Not reported	X-rays	Presence of bone regeneration and satisfactory function

CSD, critical size defect; ICBG, iliac crest bone graft; IMN, intermedullary nailing; RIA, Reamer/Irrigator/Aspirator.

There are only three studies in humans where bioabsorbable membranes have been used for reconstruction of segmental or large mandibular bone defects using bioresorbable PLLA barrier membrane (mesh) in combination with autologous bone graft (Table 6) [33,57,133]. The majority of the bone defects were secondary to benign or malignant tumors of the mandible, but other causes included infection, alveolar atrophy and trauma. Overall, the preliminary clinical results were satisfactory (rated as excellent and good in 56.5% and 27.4%, respectively). Radiologically, a certain degree of bone absorption was noted in more than half of the cases; nevertheless, only in one case was the absorption significant (up to 30%).

Finally, regarding the use of bioabsorbable membranes in long bone defects, there are only two clinical studies reporting on the clinical results in a total of 16 patients (Table 7) [13,134]. Long bone defects were mainly posttraumatic, but there were also a few cases of osteomyelitis and benign tumor resection. The bioresorbable PLLA synthetic membrane used was used in combination with autologous cancellous bone graft or bone marrow, and long bone fixation. Preliminary results showed healing of the defects and satisfactory function in all cases, except one which required further intervention.

## Discussion

Barrier membranes are among the most widely studied scaffolds for tissue regeneration, including bone, and the choice of type of membrane depends largely on the required duration of membrane function [23]. Regarding bone regeneration, their use is mainly indicated for bone regeneration in sites where limited mechanical loading exists, such as in cranial, oral and maxillofacial applications. Even though there is extensive research on barrier membranes in animals, human studies are still few. Therefore, the most reliable current evidence originates mainly from studies in animals of higher phylogenetic scale which are still limited in number. Findings from the experimental setting indicate that GBR follows the same course of steps regardless of the animal. Bone quality though is highly dependent on the species (evolution hierarchy), bone healing potential (age, general nutritional status), the membrane used, local conditions (vascularity, embryological origin of bone) and load-sharing pattern of the fixation method; and, therefore, the results and the potential clinical use should be interpreted with caution [2,13,33,76,123,130].

### Long bone versus maxillofacial bone defects

According to the preliminary clinical reports, the time period for complete regeneration of bone in the mandible is three months, whereas long bones require more than two times the same period (seven months) [33,57]. This is most likely to be attributed to the greater vascularity of the mandible and the surrounding soft tissues as well as to the different mechanical environment and less stress-shielding of the fixation method used. Furthermore, it may also be explained by the different pathways of bone formation during the regeneration process due to the different embryological origin of the mandible (intramembranous ossification) compared to long bones (endochondral ossification) [135]. Considering these differences, the 'ideal' barrier membrane may be different for maxillofacial and orthopedic applications. For example, in the case of long bone defects, the 'ideal' membrane may require improved mechanical properties, a prolonged degradation period in the case of an absorbable membrane, and even different membrane porosity to allow vascular ingrowth from the surrounding soft tissues to optimize bone formation within the defect.

### Is current evidence adequate enough for use in humans?

Despite the fact that experimental evidence is well established and preliminary results from clinical studies are encouraging, there are still several points which prevent the safe and wide use of bioabsorbable membranes in humans. Healing potential in humans is different from that of animals and it occurs with various speeds in different bones (for example, mandible versus tibia), mainly due to the difference in vascularity and/or embryological origin. Therefore, the size of the segmental defect, able to be bridged using membranes, is not yet defined in humans [132]. Additionally, the load-bearing of different bones varies widely. Even if the bone gap may be successfully bridged by the regenerated bone, more evidence is required regarding the time it will be structurally mature to cover the functional requirements. Since load-bearing is vital for the formation and progression of bone formation, the load sharing capacity of the fixation method is of utmost importance. There is no information yet on how the new bone will develop and mature in various types of fixation methods, that is, which may be considered the optimal fixation for bone regeneration in humans.

Other major parameters affecting the efficacy of bone regeneration are the characteristics of the membranes, such as composition, thickness, porosity, and perforation size [13,132]. These variables are yet to be defined in humans, because they may act in conjunction with the healing potential of each bone and may be used to optimize bone regeneration in bones with low healing potential or with a deficient local environment.

### Specific considerations for orthopedic surgery

Bioresorbable membranes are currently being used mainly for bone regeneration in oral and maxillofacial surgery in humans. However, their use in various orthopedic conditions also represents a field of interest, especially since the number of revision surgeries [136,137] and limb salvage procedures is increasing [138,139]. For example, such membranes can be shaped as tubular chambers, thus preserving the continuity of the diaphysis for the repair of large diaphyseal bone defects [140]. By forming a 'tube-in-tube implant' using two concentric perforated membranes in combination with cancellous bone-graft, the reconstitution of the 'neocortex' with well-defined thickness was possible for the treatment of segmental diaphyseal defects in sheep tibiae [132]. Barrier membranes can also help to prevent significant absorption of the bone graft which is estimated to be up to 40% to 50% at four weeks [132] and seems to be due to absorption of bone that is not mechanically functioning [141]. As these membranes are radiolucent, they allow assessment of bone formation with conventional radiographs, CT or MRI [13], which is important for monitoring the regeneration process.

The evidence on the efficacy for cortical perforation (decortication) during GBR procedures in an effort to enhance bone formation remains controversial [142]. Studies have shown that cortical perforations increase the blood supply, facilitate angiogenesis, and allow access for progenitor cells from the bone marrow into the 'chamber' [142] whereas other studies showed that bone formation occurred from a non-injured cortical bone surface and that perforations were not required as they did not increase bone formation [59,81]. However, since there are no relevant human clinical studies and the relevant animal studies refer to mandibular defects, where local vascularity is superior to long bones, recommendations for additional bone decortication cannot be made for orthopedic GBR applications [142].

Finally, barrier membranes can be used in combination with bone grafting to augment osseointegration of orthopedic implants in the case of bone defects [143]. They may also be used for regeneration of other tissues with potential orthopedic applications, including tendon regeneration in rotator cuff repair, and post-traumatic nerve regeneration [144,145], as the preliminary results are encouraging.

### Enhancement of bone regeneration and future research

#### Biological augmentation of GBR with growth factors

The interest in accelerating bone formation has led researchers to combine the membrane technique with osteoinductive or growth factors. Although the concept of additional biological enhancement of bone formation using growth factors that enhance proliferation, chemotaxis, and differentiation of osteogenic cells seems promising, results are often controversial. In a study evaluating the long-term outcome of oral implants placed in bone augmented with an allograft and a collagen membrane with or without the addition of recombinant-human bone morphogenetic protein-2 (rhBMP-2), no statistically significant differences were observed regarding the clinical and radiological outcomes [146]. On the contrary, numerous *in vivo *and *in vitro *studies have demonstrated improved bone formation when barrier membranes are loaded with platelet-derived growth factor (PDGF-BB) [147], basic fibroblast growth factor (FGF2) [148], and rhBMP-2 [99,146,149].

Controversial evidence may be secondary to insufficiency in maintaining therapeutic concentrations of growth factors within bone defects due to rapid clearance and use of different delivery methods with supraphysiological non-standarized doses to obtain therapeutic efficacy [147]. Furthermore, current research usually evaluates one or a combination of two growth factors, which does not reflect the complex physiological process of bone formation. Research is ongoing to develop novel membranes and scaffolds with improved growth factor delivery systems to accelerate bone regeneration of critically-sized segmental bone defects with promising preliminary results [150]. Moreover, with a controlled spatiotemporal delivery of growth factors, adequate local protein concentrations can be improved and maintained for optimal regenerative efficacy, avoiding the currently used supraphysiologic doses and the concomitant adverse effects [151]. Finally, the optimal 'combination' of growth factors to be delivered has also to be established.

#### Other strategies to improve bone regeneration

Aiming to maximize or accelerate bone formation, supplementary strategies have been investigated in combination with barrier membranes and grafting. The potential use of low-level laser therapy (LLLT) has been evaluated as an adjunct for the regeneration of long bone defects in animal studies with positive results [114,152]. Supplementary treatment with hyperbaric oxygen has also shown synergistic regenerative effects in the past [153]. Additionally, preliminary results have shown that systemic administration of synthetic salmon calcitonin accelerated bone regeneration of the defects [154].

Research is ongoing to evaluate other methods to enhance bone regeneration, such as local administration of parathyroid hormone (PTH(1-34)) [155] and other growth factors [156] with promising preliminary results. Moreover, methods to optimize surface microtopography of the membranes have also been investigated to enhance bone formation at the cellular and molecular level [157]. Finally, in the future, improved barrier membranes can be used as part of the bone-tissue engineering approach combined with osteoprogenitor cells and/or osteopromotive factors or even gene therapy, aiming to produce improved composite grafts [1]. Preliminary research is promising. For example, a novel three-dimensional porous polymer poly(ε-caprolactone) (PCL) scaffold coated with adeno-associated virus encoding BMP2 using both *ex vivo *or *in vivo *gene therapy, led to increased bone ingrowth with increased mechanical properties in a rat femoral defect model [158].

## Conclusions

The concept of barrier membranes for restoration of large bone defects has been developed in an effort to simplify their treatment by offering a sinlge-staged procedure and to overcome the limitations of current bone regeneration strategies. Research in this field is ongoing, with evidence being mainly gained from preclinical studies. Preliminary human studies have also shown promising results in maxillofacial, oral and orthopedic surgery. Nevertheless, before clinical applications can be recommended, future research should aim to generate and establish the 'ideal' barrier membrane. The additional use of bone-grafting materials within the membrane to fill the defect should also be evaluated, aiming to 'mimic' or even accelerate the normal process of bone formation. Finally, reproducible results and long-term observations with certified barrier membranes in animal models are required, and especially in large animal long bone defect models, as well as well-designed clinical studies to evaluate their safety, efficacy and cost-effectiveness.

## Abbreviations

ABG: autologous bone graft; ADM: acellular dermal matrix; BAMs: bioabsorbable membranes; BBM: bovine bone marrow; BCM: bovine collagen membrane; BG: bone graft; BMP: bone morphogenetic protein; BP: bovine pericardium; CAF: calcium alginate film; CaP: calcium phosphate; CM: collagen membrane; CCM: cross-linked collagen membrane; CSD: critical size defect; CT: computed tomography; DPPA: diphenylphosphorylazide; e-PTFE: expanded polytetrafluoroethylene; FGF: fibroblast growth factor; GBR: guided bone regeneration; GDF: growth differentiation factor; GTR: guided tissue regeneration; HA: hydroxyapatite; HFL: human fascia lata; HFT: human fascia temporalis; HP: human pericardium; ICBG: iliac crest bone graft; IMN: intramedullary nailing; MRI: magnetic resonance imaging; muCT: micro-computer tomography; nHA: nano-HA; PCL: poly(ε-caprolactone); PDLLCL: poly(dl-lactide-epsilon-caprolactone); PDGF: platelet-derived growth factor; PDTE: poly desaminotyrosyl-tyrosine-ethyl ester; PES: polyethersulfone; PLCL: poly lactide-co-ε-caprolactone; PLGC: poly L-lactide-co-glycolide-coepsilon- caprolactone; PLLA: poly(L-lactide) acid; PLGA: poly(L-lactide)-co-glycolide acid; PRP: platelet-rich plasma; rh-BMP: recombinant human BMP; RIA: Ria/Irrigator/Aspirator; SIS: small intestine submucosa; TCP: tricalcium phosphate.

## Competing interests

The authors declare that they have no competing interests.

## Authors' contributions

RD and GIM contributed in the preparation of this manuscript in terms of literature review and writing-up. GMC and PVG contributed in the writing of specific sections of the manuscript and in revising it critically for important intellectual content. All authors read and have given final approval of the final manuscript.

## Pre-publication history

The pre-publication history for this paper can be accessed here:

http://www.biomedcentral.com/1741-7015/10/81/prepub
